# Author Correction: Identifying inhibitors of epithelial–mesenchymal plasticity using a network topology-based approach

**DOI:** 10.1038/s41540-020-0139-7

**Published:** 2020-06-12

**Authors:** Kishore Hari, Burhanuddin Sabuwala, Balaram Vishnu Subramani, Caterina A. M. La Porta, Stefano Zapperi, Francesc Font-Clos, Mohit Kumar Jolly

**Affiliations:** 1 Centre for BioSystems Science and Engineering, Indian Institute of Science, Bangalore, 560012 India; 2 Department of Biotechnology, Indian Institute of Technology Madras, Chennai, 600036 India; 3 School of Mathematics, Indian Institute of Science Education and Research, Thiruvananthapuram, 695551 India; 4 Center for Complexity and Biosystems, University of Milan, 20133 Milano, Italy; 5 Department of Environmental Science and Policy, University of Milan, 20133 Milano, Italy; 6 Department of Physics, University of Milan, 20133 Milano, Italy

**Keywords:** Multistability, Regulatory networks, Dynamical systems, Cancer

Correction to: *npj Systems Biology and Applications* 10.1038/s41540-020-0132-1, published online 18 May 2020

The original version of this Article omitted the following the Acknowledgements:

M.K.J. was supported by Ramanujan Fellowship awarded by Science and Engineering Research Board (SERB), Department of Science and Technology (DST), Government of India (SB/S2/RJN-049/2018).

This has now been corrected in both the PDF and HTML versions of the Article.

