# Supplementary material for: Identifying inhibitors of epithelial–mesenchymal plasticity using a network topology-based approach
Source: NPJ Syst Biol Appl. 2020 May 18;6:15. doi: 10.1038/s41540-020-0132-1 (PMC7235229; doi:10.1038/s41540-020-0132-1)
Supplement: Supplementary file 2 — Supplementary [file 41540_2020_132_MOESM2_ESM.pdf]

1 Validating RACIPE simulation parameters

As mentioned in methods, we used the default simulation parameters except for the number of network parameter sets to be chosen. Among other simulation parameters, the integration method and the number of initial conditions at which each network parameter set is simulated have the potential to impact the phenotypic distributions significantly. Hence, to check the effects of these two simulation parameters, we chose networks representative of the network sizes considered in the paper, i.e, GRHL2 to represent small networks, NRF2 to represent medium sized networks and EMT_RACIPE to represent the large networks. We then simulated each of these networks in 4 ways:

- RACIPE100: RACIPE with 10000 parameter sets and default values for all other simulation parameters (specifically, 100 initial conditions per parameter set and Euler's method of integration).
- Matlab100: ode23s algorithm [1] in MATLAB R2018b (Mathworks) with 100 initial conditions and same parameter sets as obtained from RACIPE100 simulations. Since ode23s is a stiff system solver, this simulation gives us the effect of stiff systems the can potentially arise due to random sampling of network parameter sets.
- Matlab1000: Matlab ode23s with 1000 initial conditions and same parameter sets as above. This, in comparison with the previous simulations, reflects the effect of number of initial conditions used to obtain the steady states.
- RACIPE1000: RACIPE with 1000 simulations to check the effects of initial conditions in the operating domain of RACIPE. Since the RACIPE realization here is different from RACIPE100 simulations, the parameter sets need not be the same.

In Matlab simulations, the steady state was said to have reached when state change over 10 time-steps was less than 0.001. All the simulations were run in triplicates. We then compared the discretized phenotypic frequencies from these simulations in pairs, and measured:

- Fold change in the phenotype frequency levels using volcano plots, with p-values obtained from t-test on n=3 replicates. The null hypothesis is that the mean log fold change is 0.
- JSD of mean phenotypic distributions.

The results, shown in **Fig S7**, suggest that while these simulation parameters can affect frequencies of individual phenotypes, the changes at an ensemble level, as measured by JSD, are minute.

References

[1] Shampine, L. F. & Reichelt, M. W. The MATLAB ODE suite. *SIAM Journal on Scientific Computing* **18**, 1–22 (1997).

2 Supplementary Tables

Table 1: GRHL2 single-edge perturbations including edge-additions. A given single edge perturbation is named as : FromNode-ToNode_OriginalEdge-PerturbedEdge. 0 : no edge, 1: activation, 2: inhibition. Network number 2 is 'wild-type'

Label	Network
1	GRHL2-GRHL2_0-1
3	ZEB-miR200_2-1
4	GRHL2-GRHL2_0-2
5	miR200-miR200_0-1
6	miR200-ZEB_2-0
7	miR200-ZEB_2-1
8	SNAIL-SNAIL_0-1
9	ZEB-miR200_2-0
10	ZEB-ZEB_1-0
11	ZEB-ZEB_1-2
12	ZEB-GRHL2_2-0
13	ZEB-GRHL2_2-1
14	SNAIL-GRHL2_0-1
15	SNAIL-GRHL2_0-2
16	GRHL2-ZEB_2-0
17	GRHL2-ZEB_2-1
18	SNAIL-ZEB_1-2
19	GRHL2-miR200_0-1
20	GRHL2-SNAIL_0-2
21	GRHL2-miR200_0-2
22	GRHL2-SNAIL_0-1
23	SNAIL-ZEB_0-1
24	miR200-GRHL2_0-1
25	SNAIL-ZEB_0-2
26	SNAIL-ZEB_1-0
27	miR200-GRHL2_0-2
28	miR200-miR200_0-2
29	miR200-SNAIL_0-1
30	miR200-SNAIL_0-2
31	SNAIL-miR200_2-0
32	SNAIL-miR200_2-1

Table 2: NRF2 perturbations without edge-additions. A given single edge perturbation is named as : FromNode-ToNode_PreviousEdge-OriginalEdge. 0 : no edge, 1: activation, 2: inhibition. Network number 11 is 'wild-type'

Label	Network
1	Keapl-NRF2_2-0
2	E-cad-NRF2_2-1
3	ZEB-ZEB_1-2
4	ZEB-miR34_2-0
5	miR200-Keap1_2-0
6	ZEB-miR200_2-0
7	SNAIL-miR34_2-0
8	SNAIL-ZEB_1-2
9	miR34-SNAIL_2-0
10	SNAIL-SNAIL_2-0
12	ZEB-miR200_2-1
13	miR200-ZEB_2-1
14	NRF2-SNAIL_2-1
15	E-cad-ZEB_2-1
16	ZEB-ZEB_1-0
17	miR200-Keapl_2-1
18	SNAIL-miR34_2-1
19	ZEB-E-cad_2-1
20	E-cad-NRF2_2-0
21	I-SNAIL_1-2
22	E-cad-ZEB_2-0
23	ZEB-E-cad_2-0
24	miR200-ZEB_2-0
25	SNAIL-miR200_2-0
26	SNAIL-SNAIL_2-1
27	SNAIL-miR200_2-1
28	NRF2-SNAIL_2-0
29	SNAIL-ZEB_1-0
30	Keapl-NRF2_2-1
31	miR34-SNAIL_2-1
32	ZEB-miR34_2-1

3 Supplementary Figures

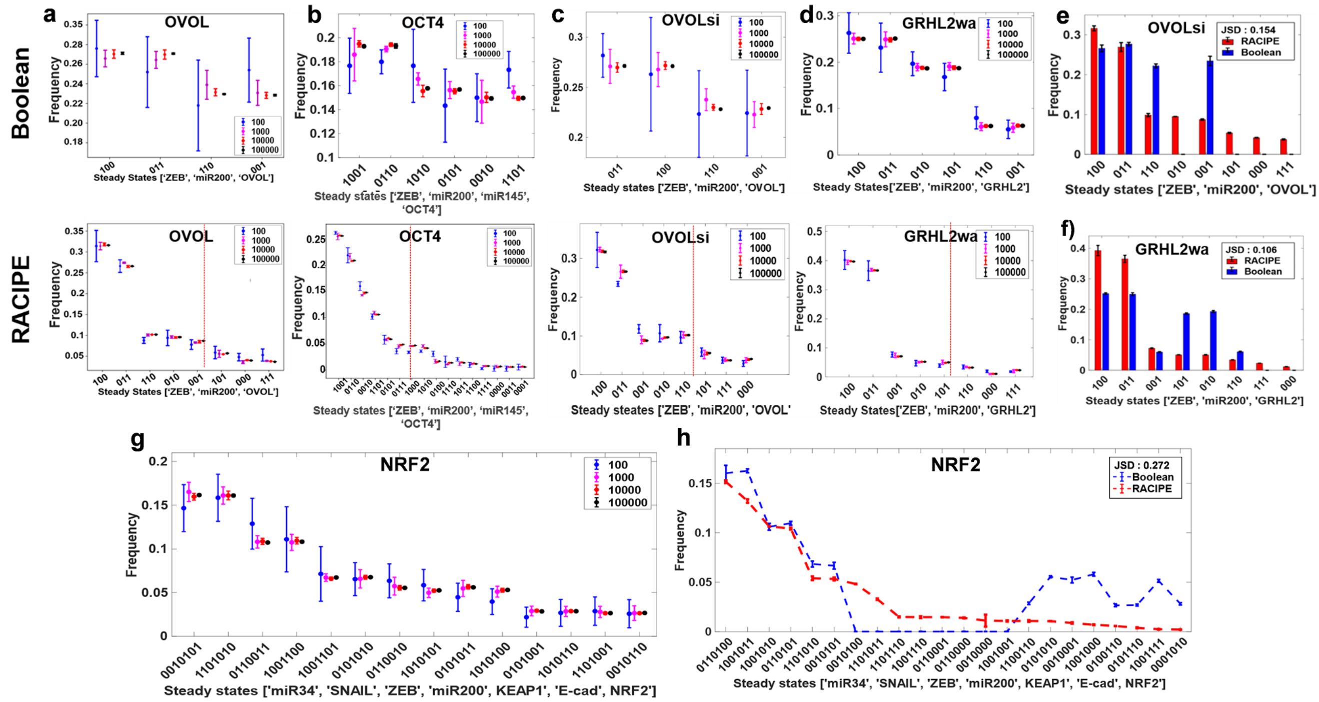

Figure S1: Quantitative convergence (QC) and comparison of phenotypic distributions obtained from RACIPE and Boolean QC of **a** OVOL, **b** OCT4, **c** OVOLsi and **d** GRHL2wa using Boolean (top) and RACIPE (bottom). **e-f.** Comparision of steady state frequencies obtained from RACIPE and Boolean for GRHL2wa and OVOLsi **g-h.** NRF2 QC plot (Boolean) and comparison of RACIPE and Boolean state frequency distributions

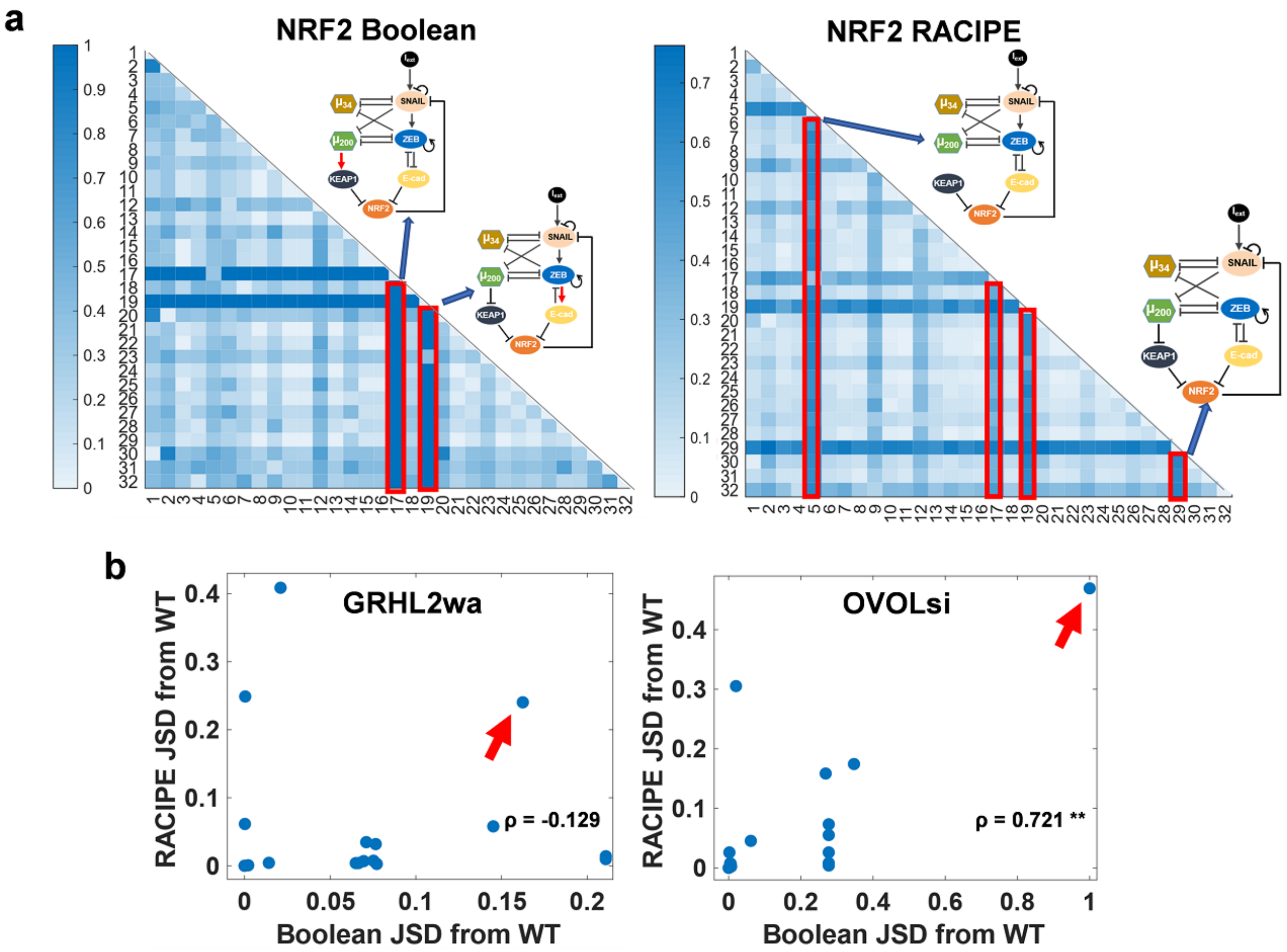

Figure S2: Effect of perturbations on phenotype distribution **a.** Heatmap of JSD of perturbations made to NRF2 network from each other (no edge additions) as obtained from i) Boolean and ii) RACIPE. The strongest perturbations are highlighted and the corresponding networks are shown with the heatmap. **b** Scatter plots of JSD of perturbed networks from WT obtained from Boolean and RACIPE for GRHL2wa and OVOLsi.

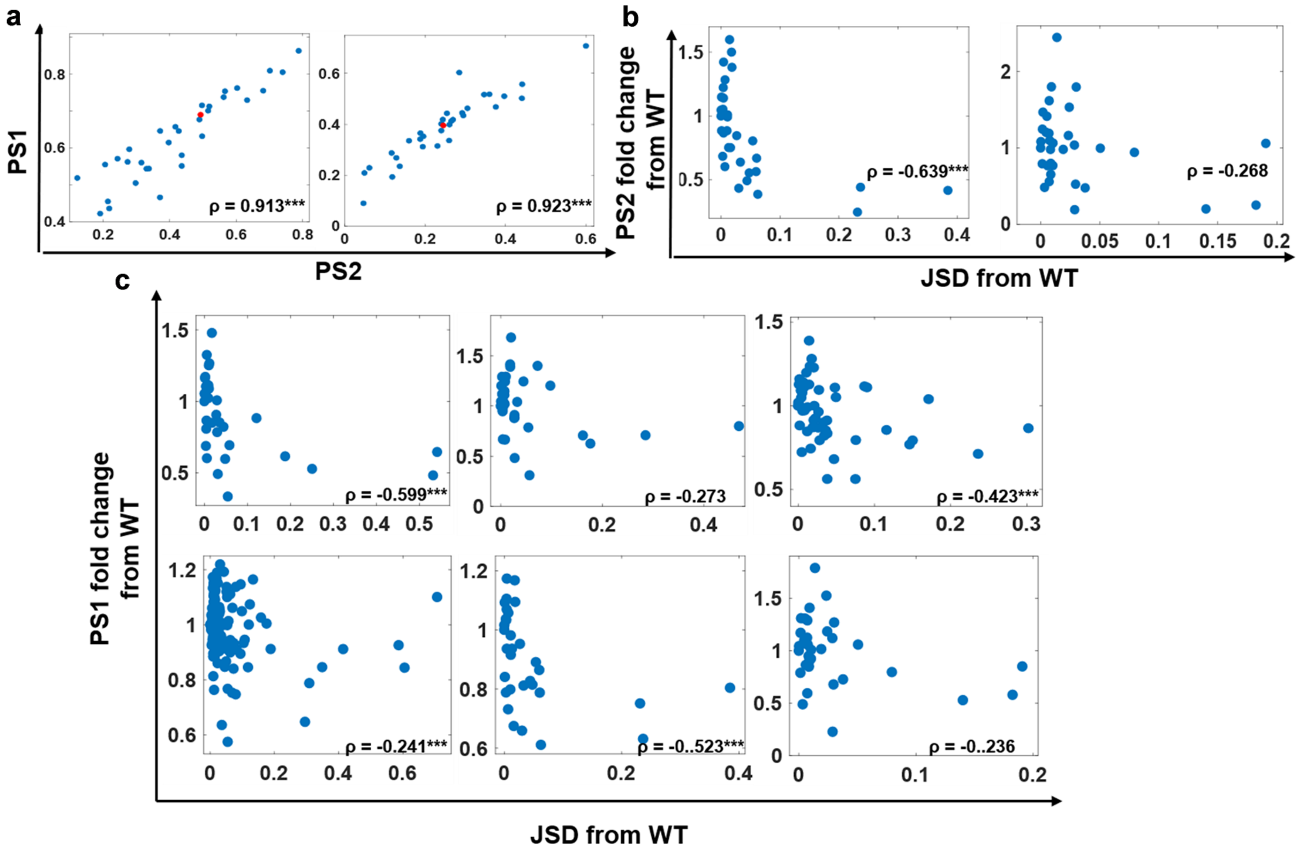

Figure S3: Effect of JSD on phenotypic plasticity **a.** Scatter plot between PS1 and PS2 for GRHL2wa and OVOLsi. **b.** Scatter plots for GRHL2wa and OVOLsi between fold change in plasticity vs change in phenotypic distribution (JSD). Each dot represents a perturbed network topology of the mentioned network. **c.** Same plots as B for all the networks, using PS1 instead of PS2

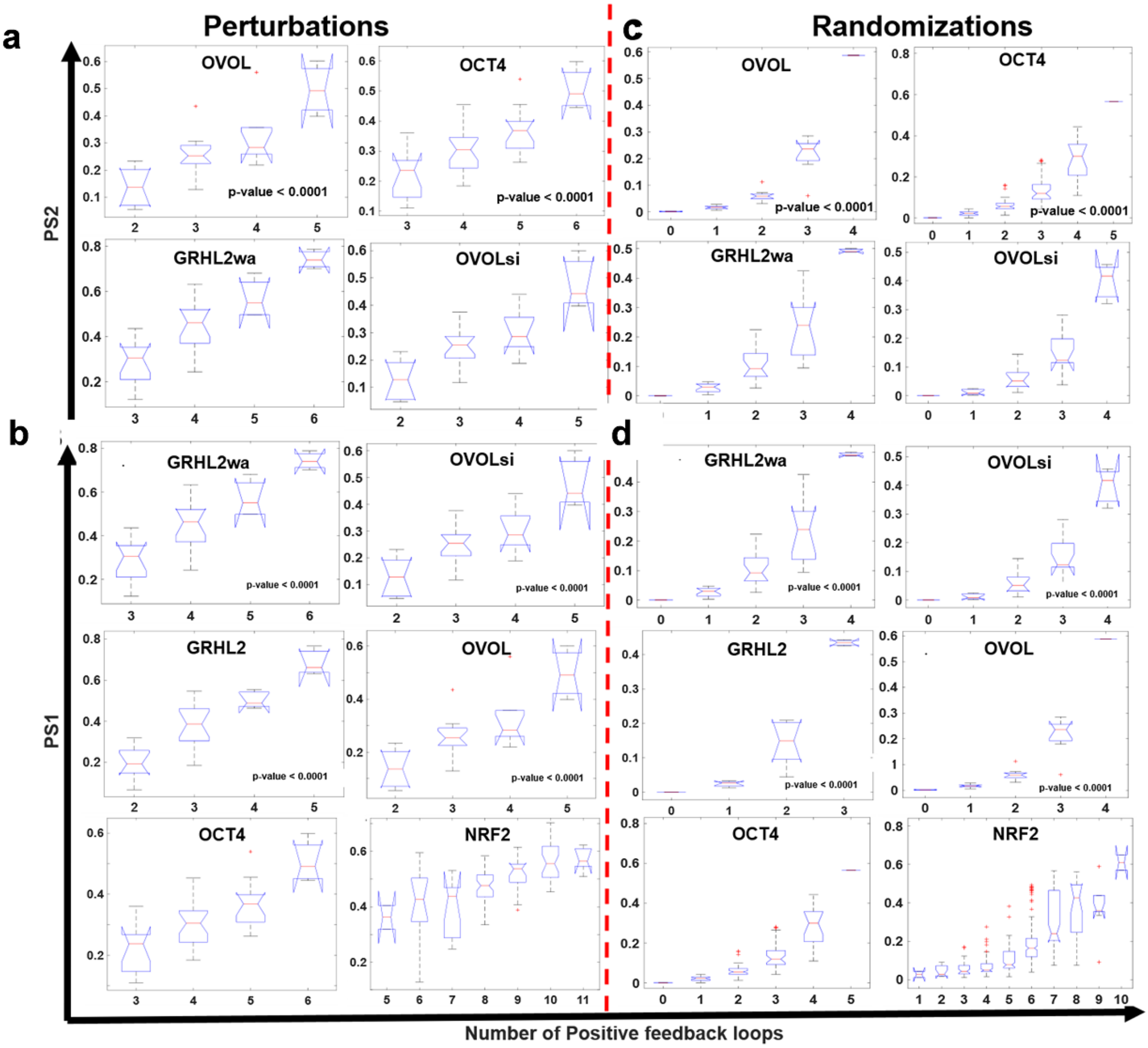

Figure S4: Effect of positive feedback loops on plasticity **a.** Box-plot of PS2 vs positive cycles for GRHL2wa and OVOLsi perturbed network topologies **b.** Box-plots of PS1 vs positive cycles for all perturbed network topologies coresponding to each EMP network **c.** Same as A but randomized circuits **d.** Same as B but randomized circuits

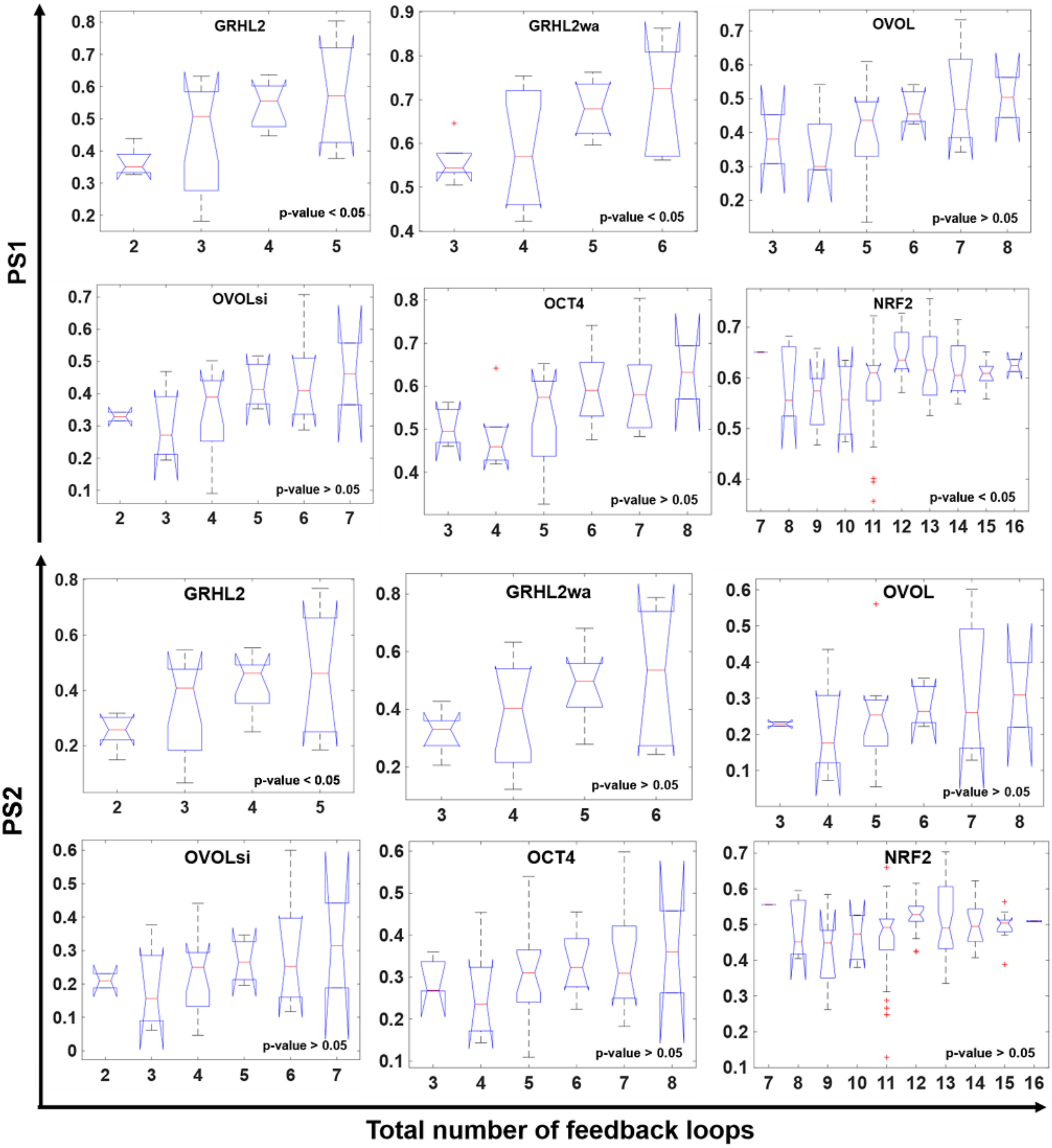

Figure S5: Effect of total number of feedback loops on network plasticity for perturbations of all 6 networks.

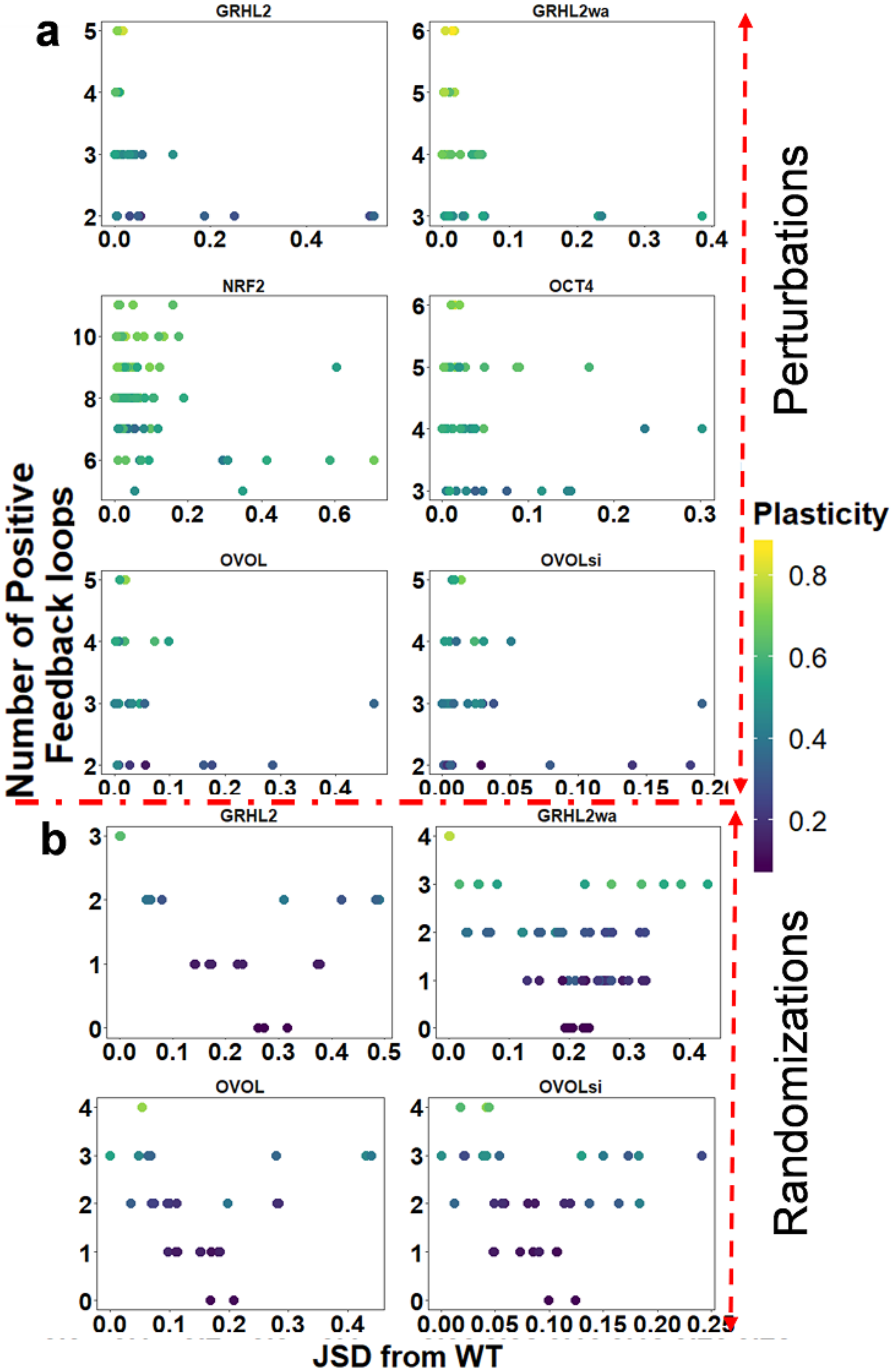

Figure S6: Combined effect of positive cycles and JSD on plasticity A. Positive cycles vs JSD scatter plots for perturbed network topologies of all 6 networks, colored according to plasticity B. Same as A but for randomized circuits

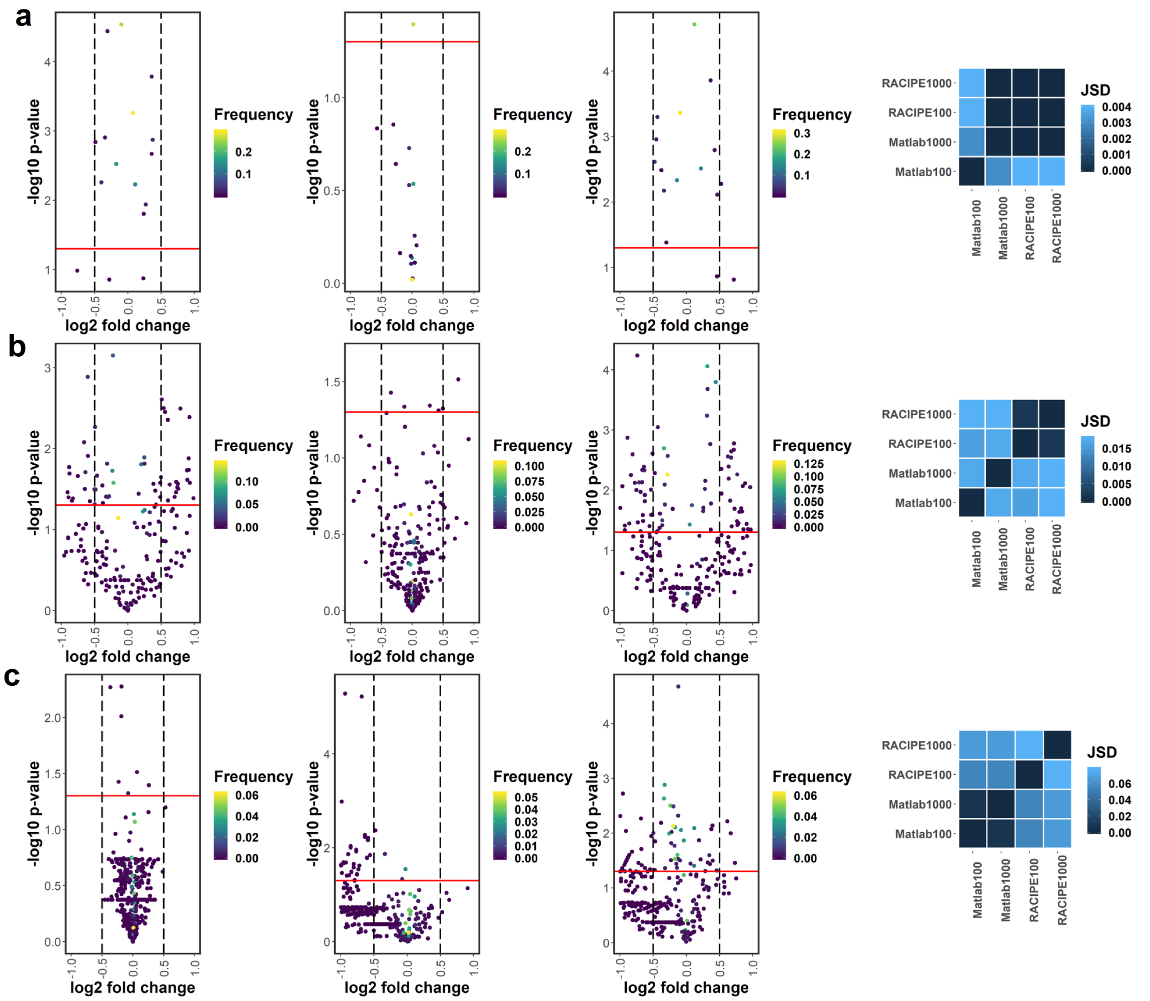

Figure S7: Testing RACIPE simualtion parameters. **a.** Results for GRHL2. Volcano plots compare fold changes in steady states from Matlab100 and MATLAB1000 (left), RACIPE100 and RACIPE 1000 (middle), RACIPE100 and MATLAB100 (right). Each point represents a steady state. The red horizontal line corresponds to p=0.05. Fold change in any steady state below the red line is insignificant. The vertical lines correspond to a fold change of 0.7 and 1.41 respectively. Each steady state has been colored according its observed frequency. JSD heatmap of the mean phenotypic distributions obtained from all 4 simulations (right most panel). **b.** Same as A but for NRF2. **c.** Same as A but for EMT_RACIPE
